# A Melting Curve-Based Multiplex RT-qPCR Assay for Simultaneous Detection of Four Human Coronaviruses

**DOI:** 10.3390/ijms17111880

**Published:** 2016-11-23

**Authors:** Zhenzhou Wan, Ya’nan Zhang, Zhixiang He, Jia Liu, Ke Lan, Yihong Hu, Chiyu Zhang

**Affiliations:** 1 Pathogen Diagnostic Center, CAS Key Laboratory of Molecular Virology & Immunology, Institute Pasteur of Shanghai, Chinese Academy of Sciences, Shanghai 200025, China; wanlv@126.com (Z.W.); 13901433616@139.com (Y.Z.); kevin2003xzyz@163.com (Z.H.); liujia02@sibs.ac.cn (J.L.); lanke@sibs.ac.cn (K.L.); 2 Medical Laboratory of Taizhou Fourth People’s Hospital, Taizhou 225300, China

**Keywords:** human coronaviruses, melting curve, multiplex quantitative RT-PCR (RT-qPCR), SYTO 9, melting temperature (Tm)

## Abstract

Human coronaviruses HCoV-OC43, HCoV-229E, HCoV-NL63 and HCoV-HKU1 are common respiratory viruses associated with acute respiratory infection. They have a global distribution. Rapid and accurate diagnosis of HCoV infection is important for the management and treatment of hospitalized patients with HCoV infection. Here, we developed a melting curve-based multiplex RT-qPCR assay for simultaneous detection of the four HCoVs. In the assay, SYTO 9 was used to replace SYBR Green I as the fluorescent dye, and GC-modified primers were designed to improve the melting temperature (Tm) of the specific amplicon. The four HCoVs were clearly distinguished by characteristic melting peaks in melting curve analysis. The detection sensitivity of the assay was 3 × 10^2^ copies for HCoV-OC43, and 3 × 10^1^ copies for HCoV-NL63, HCoV-229E and HCoV-HKU1 per 30 μL reaction. Clinical evaluation and sequencing confirmation demonstrated that the assay was specific and reliable. The assay represents a sensitive and reliable method for diagnosis of HCoV infection in clinical samples.

## 1. Introduction

The human coronaviruses (HCoVs) are enveloped, nonsegmented positive strand RNA viruses. They belong to the family coronaviridae, genus coronavirus [1,2]. Their genomes are 27–31 kb in length. The 5′ end of their genome contains two large open reading frames (ORFs), ORF1a and ORF1b, encoding the replicase complex. Genes encoding the structural proteins, i.e., spike (S), envelope (E), membrane (M) and nucleocapsid (N) proteins, are downstream of the ORF1b [1]. Six coronaviruses (HCoV-229E, HCoV-OC43, SARS-CoV, HCoV-NL63, HCoV-HKU1, and MERS-CoV) are known to infect humans [2]. HCoV-229E and HCoV-OC43 were identified in the 1960s [3,4]. HCoV-NL63 and HCoV-HKU1 were identified in 2004 and 2005, respectively [5,6]. SARS-CoV was identified during the severe acute respiratory syndrome (SARS) epidemic in China in 2003 [7,8], and MERS-CoV was identified during the Middle East respiratory syndrome (MERS) epidemic in the Middle East in 2012 [9].

Among these HCoVs, HCoV-OC43, HCoV-229E, HCoV-NL63 and HCoV-HKU1 are kinds of most commonly observed respiratory viruses [2], which are often associated with children with acute respiratory infection. The four HCoVs are distributed globally with various prevalence rates (1.6%–16%) in different countries/regions [10,11,12,13]. The positive rate was about 2.6% in all ages [10]. The infection of the four HCoVs was associated with a range of respiratory symptoms (e.g., fever, cough, rhinorrhea, bronchitis, etc.) and often led to mild, self-limiting disease [14]. Only few cases with infection of the four HCoVs were associated with pneumonia requiring hospitalization, and even fatal outcomes [15,16]. Furthermore, infection with the four HCoVs might be also involved in digestive dysfunctions [17]. There is no efficient vaccine available for the prevention of infection with the four HCoVs [17]. Therefore, the development of rapid and sensitive assay for the monitoring of HCoV infection is important for the management and treatment of hospitalized patients with HCoV infection.

Early methods for the detection of the four HCoVs included serologic analysis, virus culture, and/or antigen detection [18,19,20]. However, these methods were often time-consumingand low sensitive, and easily caused cross-reaction with other HCoVs [20,21,22,23]. Relative to these conventional methods, PCR-based detection methods are more sensitive, specific, rapid and simple [21,24]. Recently, several multiplex RT-PCR and multiplex quantitative RT-PCR (RT-qPCR) assays were developed for detection of common human respiratory viruses, including the four HCoVs [25,26,27,28,29,30,31,32]. In the multiplex RT-PCR assay, the result was judged based on the amplicon size of corresponding respiratory virus in gel electrophoresis. Because amplicons often are designed at the range of 250–600 base pairs, the assay has a low discrimination capability and often causes misdiagnosis especially when the difference in amplicon sizes is small [25,26]. In addition, the post-PCR gel electrophoresis detection is easy to cause cross-contamination. A multiplex RT-qPCR assay was previously developed for detection of four HCoVs [12]. It not only improved the detection efficiency of four HCoVs, but also avoided the contamination of PCR products in the post-PCR processing (e.g., gel electrophoresis) [12].

Compared with hydrolysis probe-based qPCR, the fluorescent dye (e.g., SYBR green, CYTO-dye)-based assay is cheaper. In the present study, we developed a melting curve-based multiplex RT-qPCR assay for the detection of four HCoVs (HCoV-229E, -OC43, -NL63, and -HKU1). In the assay, SYTO 9 was used to replace SYBR Green I as the fluorescent dye, and GC-modified HCoV-OC43 primers were designed to improve the melting temperature (Tm) of PCR amplicon. The four HCoVs can be detected and distinguished by specific melting peaks.

## 2. Results

### 2.1. Selection of Primer Sets, and Modification of OC43 Primers

**Figure 1:**
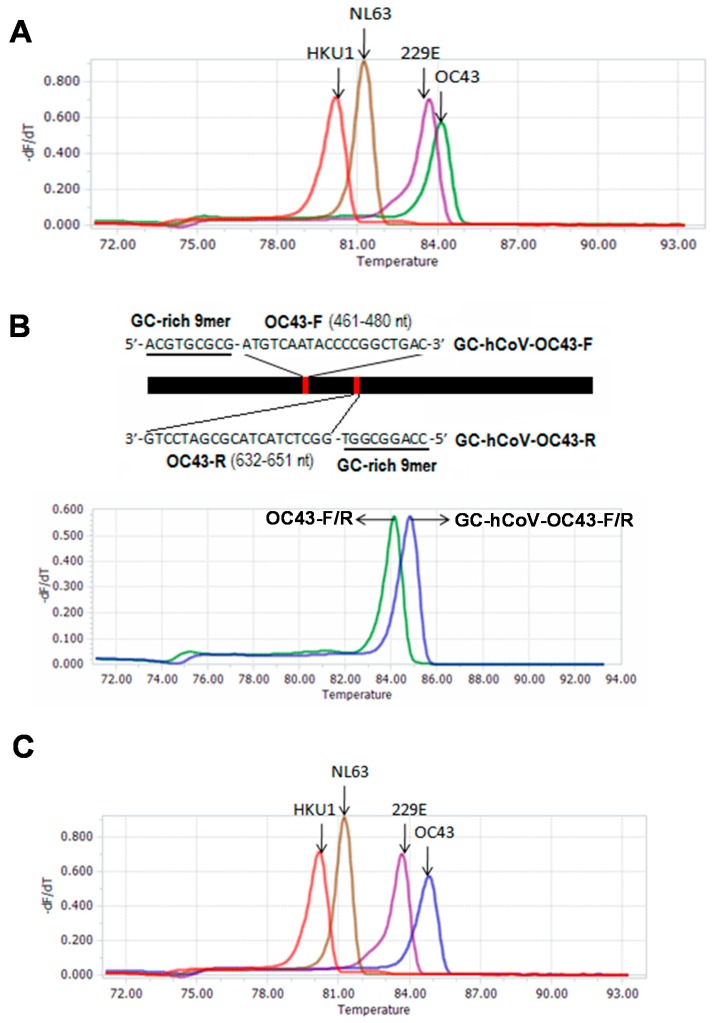
Melting curves of four HCoVs using the multiplex RT-qPCR assay. (**A**) The melting curves generated using first set of primers of each HCoV; (**B**) Improvement of Tm value of OC43 amplicon using GC-modified HCoV-OC43 primers; (**C**) The melting curves generated using the optimized primer sets with GC-modified HCoV-OC43 primers for four HCoVs.

To develop a melting curve-based multiplex RT-qPCR assay for simultaneous detection of four human coronaviruses (HCoV-OC43, -229E, -NL63 and -HKU1), we designed 10 sets of primers for amplification of the four HCoVs. The theoretical Tm values for 10 amplicons were from 81.44 to 84.97 °C (Table 1). In order to select the optimal combination of the primer sets for the four HCoVs, the actual Tm values of 10 specific amplicons were measured by a melting curve analysis. The actual Tm values were from 80.00 to 83.96 °C (Table 1). The difference between theoretical and actual Tm values varied from 0.01 to 1.01 °C. To correctly distinguish the four HCoVs, the primer set 1 of each HCoV was selected to combine into a detection group (Figure 1A). The corresponding Tm values were 83.39, 83.96, 81.10, and 80.00 for HCoV-229E, HCoV-OC43, HCoV-NL63, and HCoV-HKU1, respectively (Figure 1A). Although HCoV-229E, -NL63, and -HKU1 can be clearly distinguished from each other by Tm value, the small difference in Tm values (0.57 °C) between amplicons of HCoV-229E and HCoV-OC43 may result in weak discrimination of both HCoVs.

To improve the discrimination between HCoV-229E and HCoV-OC43 amplicons, we developed a strategy to increase the Tm value of the HCoV-OC43-specfic amplicon by modifying the specific primers. First, we generated a series of GC-rich 9-mer oligonucleotides using the shuffle program in the online software package SMS (http://www.bioinformatics.org/SMS/), and added the GC-rich 9-mer oligos into the 5′ end of the HCoV-OC43 primers. Then, we evaluated these GC-modified HCoV-OC43 primers using Vector NTI (Version 11.5.4; Thermo Fisher Scientific Inc., Bedford, MA, USA; http://www.thermofisher.com/cn/zh/home/life-science/cloning/vector-nti-software/vector-nti-advance-software/vector-nti-advance-downloads.html) and melting curve analysis (data not shown). One set of GC-modified HCoV-OC43 primers was selected because the Tm value of the corresponding amplicon was improved from 83.96 to 84.88 °C (Figure 1B). The GC-modified HCoV-OC43 primers are GC-HCoV-OC43-F: **ACGTGCGCG**ATGTCAATACCCCGGCTGAC and GC-HCoV-OC43-R: **CCAGGCGGT**GGCTCTACTACGCGATCCTG (Figure 1B). Compared with the original OC43-F/R primers, the GC-HCoV-OC43-F/R primers also appeared to improve the amplification reactions (Figure S1). When the GC-HCoV-OC43-F/R primers were added into the detection group by replacing the original OC43-F/R primers, four amplicons of HCoVs were clearly distinguished from each other by melting peaks (Figure 1C). The corresponding Tm values were 84.88, 83.39, 81.10, and 80.00 for HCoV-OC43, -229E, -NL63, and -HKU1, respectively. These imply the establishment of a melting curve-based quadruplex RT-qPCR for detection of the four HCoVs. To obtain well performance, the primer concentration of the assay was optimized as 0.8 μM, 0.8 μM, 0.8 μM and 0.2 μM of HCoV-229E, -NL63, -HKU1, and -OC43 specific primers, respectively, by orthogonal experiments (Supplemental result and Figure S2).

### 2.2. Specificity of the Melting Curve-Based Multiplex RT-qPCR

**Figure 2:**
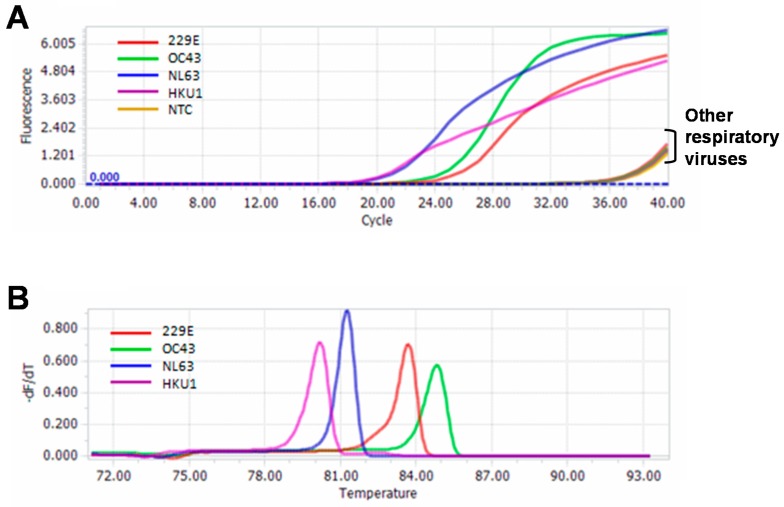
Specificity of the multiplex RT-qPCR assay. (**A**) Amplification curves of 13 common respiratory viruses; (**B**) Melting curves of 13 common respiratory viruses. NTC, non-template control.

The specificity of the melting curve-based multiplex RT-qPCR was assessed using 13 common respiratory viruses. Except the four HCoVs, no amplification was observed for other 9 respiratory viruses, as well as the negative control (Figure 2A). Melting analysis showed that except the four HCoVs specific melting peaks, there was no melting peak for other 9 respiratory viruses and negative control (Figure 2B). Furthermore, there was also no cross-amplification among these four HCoVs. These indicated that the assay had good specificity for detection of the four HCoVs.

### 2.3. Sensitivity of the Melting Curve-Based Multiplex RT-qPCR

**Figure 3:**
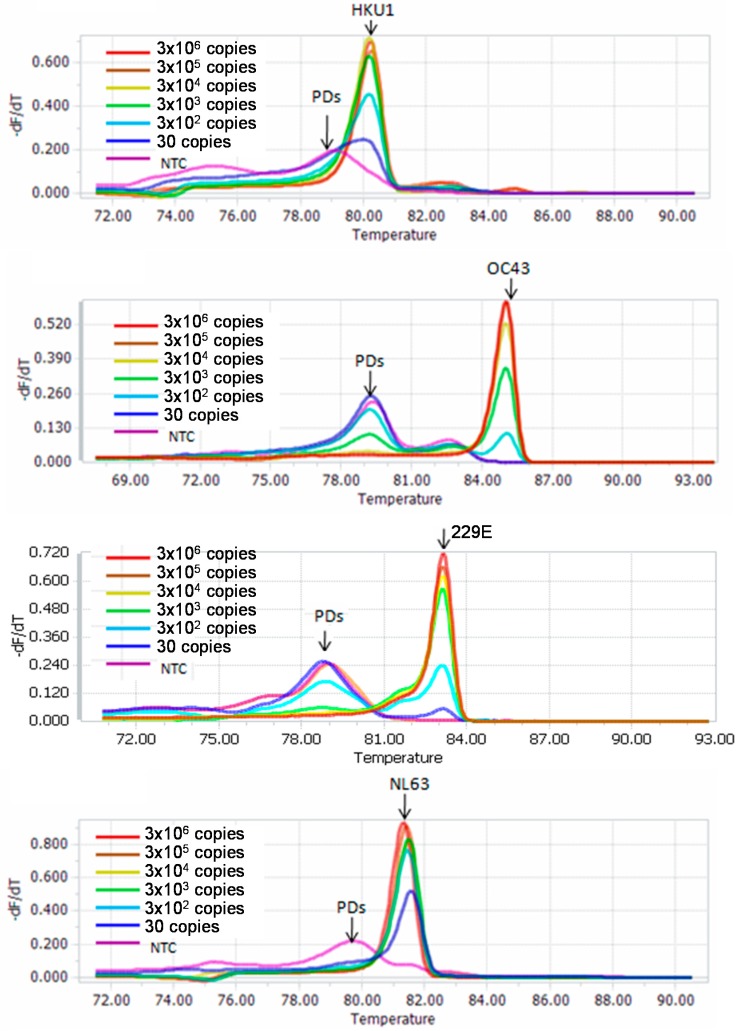
Sensitivity of the multiplex RT-qPCR assay using serially diluted RNA stocks. PDs, primer dimers; NTC, non-template control.

Sensitivity of the melting curve-based multiplex RT-qPCR was accessed using serially diluted RNA stocks for the four HCoVs from 1 × 10^6^ to 1 × 10^1^ copies/μL. The new assay detected 3 × 10^1^ copies of HCoV-NL63, -229E and -HKU1, and 3 × 10^2^ copies of HCoV-OC43 per 30 μL reaction (Figure 3). For the four HCoVs, the calibration curves had high linear relation with *R*^2^ of 0.98 to 1.0. The amplification efficiencies of the new assay for HCoV-229E, -NL63, -HKU1, and -OC43 were 0.86, 0.85, 0.84, and 1.07, respectively (Figure S3).

### 2.4. Evaluation and Confirmation of the Melting Curve-Based Multiplex RT-qPCR by Sequencing

Among 88 clinical samples, 61 were identified as HCoV-positive, including 52 single infections with one of the four HCoVs and 9 dual-infections with two of the four HCoVs. The detection rate of HCoVs was 69.3%, obviously higher than that (33.0%: 29/88) of the multiplex RT-PCR method used in our previous study. Single infections included 11 HCoV-229E, 22 HCoV-OC43, 16 HCoV-NL63, and 3 HCoV-HKU1. The proportion of dual-infection was 14.8% (9/61), and the dual-infection cases contained 5 HCoV-229E/-OC43, 2 HCoV-229E/-NL63, and 2 HCoV-NL63/-OC43.

From 61 HCoVs positive clinical samples identified by the melting curve-based multiplex RT-qPCR, we selected 21 samples having relatively strong melting peak for sequencing confirmation. Blast using the obtained sequences as query sequence revealed there were 2 HCoV-229E, 9 HCoV-OC43, 9 HCoV-NL63, and 1 HCoV-HKU1 sequences (Figure S4). Each obtained sequence corresponded to the right HCoV as judged by the melting curve-based multiplex RT-qPCR assay (Figure S5), indicating a 100% specificity at least for these sequenced samples.

## 3. Discussion

Because HCoVs are one of the most commonly observed pathogens causing respiratory illnesses [2,10,11], the development of reliable and rapid detection method is greatly needed. The multiplex RT-qPCR assay has high sensitivity and specificity, and was often used in the detection of respiratory viruses [27,28,29,30]. A hydrolysis probe-based multiplex RT-qPCR assay was previously developed for HCoV detection [12]. However, difficulty in probe design for highly divergent viruses, as well as high cost in probe synthesis, limits the use of probe-based multiplex RT-qPCR assay.

As an alternative to hydrolysis probes, fluorescent dyes (e.g., SYBR geen I) that bind preferentially to double-stranded DNA (dsDNA) have been widely used in the qPCR because they are cheaper than hydrolysis probe. However, because the binding of fluorescent dyes to dsDNA lacks sequence specificity, they are rarely used in the multiplex qPCR. Recently, several melting curve-based multiplex RT-qPCR assays using fluorescent dye were developed for simultaneous detection of bacterial pathogens [33,34,35]. Up to our knowledge, no similar method was used for detection of human viruses. In this study, we developed a melting curve-based multiplex RT-qPCR assay for the detection of four HCoVs (HCoV-229E, -OC43, -NL63, and -HKU1). Because SYBR Green I inhibits PCR reaction and preferentially binds to GC rich DNA sequences, which can affect the melting curve analysis [36], we used fluorescent dye SYTO 9 to replace SYBR Green I in the assay. In order to correctly distinguish the four HCoVs in a single RT-qPCR reaction, we designed four sets of primers to generate four specific PCR products with obviously different Tm values (Table 1). The melting curve analysis showed that the Tm values of the specific products for HCoV-229E, -OC43, -NL63, and -HKU1 were 83.39 °C, 83.96 °C, 81.10 °C, and 80.00 °C, respectively (Figure 1A). To further increase the discrimination between HCoV-229E and HCoV-OC43, we developed a strategy to improve the Tm value of HCoV-OC43 amplicon by introducing a 9-mer GC-rich oligonucleotide into the 5′ end of the specific primers. The GC-modified OC43 primers generated an amplicon with Tm value of 84.88 °C, 0.92 °C higher than that obtained with the original OC43 primers (Figure 1B). Under the new primer sets, the melting curve-based multiplex RT-qPCR assay can well detect and distinguish the four HCoVs (Figure 1C).

The detection sensitivity of the new assay was determined using the standard strains or in vitro transcribed RNA of the four HCoVs. The detection sensitivity was 3 × 10^2^ copies/μL for HCoV-OC43, and 3 × 10^1^ copies/μL for HCoV-NL63, HCoV-229E and HCoV-HKU1 (Figure 3). The specificity of the assay was assessed using a panel of respiratory viruses commonly found in children with acute respiratory infection. No amplification signal was observed for any of the strains except the four targeted HCoVs (Figure 2). Using the new assay, we detected 88 clinical samples. Among them, 61 were identified as HCoV-positive, including 9 dual infections with two of the four HCoVs. To confirm the results, we selected 21 samples (including 9 HCoV-OC43, 9 HCoV-NL63, 2 HCoV-229E and 1 HCoV-HKU1) from these positive samples for sequencing confirmation. The sequence and blast results of the 21 samples were completely consistent with that obtained by the melting curve-based multiplex RT-qPCR assay (Figures S4 and S5). Furthermore, non-specific amplification was not observed when pooled samples were used (data not shown). These results indicated that the new assay was reliable.

In summary, we developed a melting curve-based multiplex RT-qPCR assay for simultaneous detection of HCoV-229E, HCoV-OC43, HCoV-NL63 and HCoV-HKU1. The assay is a sensitive and reliable method for screening of HCoV infection in clinical samples (e.g., nasopharyngeal swabs). In addition, we developed a strategy to improve the Tm value of amplicon by adding GC-rich oligonucleotides to the 5′ end of the related primers.

## 4. Materials and Methods

### 4.1. Virus Strains and Patient Samples

Nine standard virus strains, including HCoV-OC43 (VR-1558), HCoV-229E (VR-740), human respiratory syncytial virus (hRSV)-A (VR-26) and -B (VR-1580), Influenza A (VR-99) and B (VR-789), parainfluenza type 3 (PIV3) (VR-93), rhinovirus (VR-1162), and adenovirus (VR-930), were used in this study. These virus strains were all purchased from the American Type Culture Collection (ATCC) (Manassas, VA, USA) and cultured according to ATCC recommended conditions.

A total of 88 nasopharyngeal swabs from outpatient children with fever and respiratory symptoms in Shanghai Nanxiang Hospital were used in this study for clinical evaluation. The samples were obtained from two previous studies with 59 being identified as HCoV-positive using a multiplex RT-PCR [37,38]. During this study, these samples were re-detected using the same method. Only 29 were identified as HCoV-positive, which may be ascribed to degradation of viral RNA among these samples caused by multiple freezing and thawing. The study was approved by the Ethics Committee of Taizhou Fourth People’s Hospital (No. 201602; 25 February 2016).

RNA was extracted from 140 μL of standard virus strains or clinical specimens and eluted in 60 μL Nuclease-free H_2_O using the QIAamp Viral RNA Mini Kit (Qiagen, Venlo, The Netherlands) according to the manufacturer’s instructions.

### 4.2. Primer Design

All available genomic sequences of HCoV-229E, HCoV-OC43, HCoV-NL63, and HCoV-HKU1 were downloaded from GenBank, and aligned using MEGA 5.05 (http://www.megasoftware.net/) to find the most conserved region. The *M* gene of HCoV-229E, and *N* genes of HCoV-OC43, HCoV-NL63, and HCoV-HKU1 were selected as target region for primer design. The primers were designed using Primer Express 3.0 software (Applied Biosystems, Foster City, CA, USA), and their specificity was confirmed by a Nucleotide BLAST search. In order to develop a multiplex RT-qPCR assay based on melting analysis for simultaneous detection of the four HCoVs, the primers were designed to generate amplicons with different melting temperature (Tm) for different coronavirus. Three sets of primers were designed for HCoV-229E, -OC43, and -HKU1, and one set of primers was for HCoV-NL63 (Table 1). The theoretical Tm of each amplicon was predicted using the online tool Oligo Calc: Oligonucleotide Properties Calculator (Northwestern University, Chicago, IL, USA), (http://biotools.nubic.northwestern.edu/OligoCalc.html) based on the amplicon sequence [39]. Actual Tm of the amplification product was determined by the melting curve analysis.

### 4.3. RT-qPCR and Melting Curve Analysis

The RT-qPCR was performed using QIAGEN OneStep RT-PCR Kit (QIAGEN, Hilden, Germany) with SYTO 9 (Life technologies, Carlsbad, CA, USA) as the fluorescent dye. Thirty μL reactions including 3 μL template input were run on a Light Cycler 96 RT-qPCR System (Roche Diagnostics, Mannheim, Germany). Reaction conditions were: 30 min RT at 50 °C, 15 min at 94 °C for inactivation of reverse transcriptase (RT), followed by 40 cycles of 94 °C for 30 s, 50 °C for 30 s and 72 °C for 1 min. Melting curve analysis was performed under the condition of 95 °C for 60 s, 40 °C for 60 s, 65 °C for 1 s, then followed by a slow increase from 65 °C to 95 °C with a speed of 0.07 °C per second.

### 4.4. Specificity and Sensitivity of the Melting Curve-Based Multiplex RT-qPCR

The specificity of the melting curve-based multiplex RT-qPCR was assessed using 13 common respiratory viruses including 9 standard strains and 4 clinical isolates. The 9 standard strains were described above and the 4 clinical isolates were PIV1, PIV2, PIV4, and HCoV-NL63. Because there was no standard strain and clinical isolate available for HCoV-HKU1, we synthesized plasmid containing HCoV-HKU1 *N* gene downstream of the T7 promoter. HCoV-HKU1 RNA was obtained via in vitro transcription. Using the same strategy, we also obtained the RNA stocks of HCoV-229E, HCoV-OC43, and HCoV-NL63 for sensitivity experiments. Serially diluted RNA stocks for four HCoVs from 1 × 10^6^ to 1 × 10^1^ copies/μL were used to determine the detection limit of the melting curve-based multiplex RT-qPCR.

### 4.5. Evaluation of the Melting Curve-Based Multiplex RT-qPCR Using Clinical Samples

A total of 88 clinical samples were used to evaluate the performance of the melting curve-based multiplex RT-qPCR for four HCoVs detection. To avoid the possibility of the non-specific PCR product with same or similar Tm value to specific products, we confirmed the detection results by sequencing the amplicons. Because the weak melting peak implies few amplification products that are not enough for direct DNA sequencing, we selected the amplification products with strong melting peaks for sequencing confirmation. Of HCoV-positive clinical samples identified by the multiplex RT-qPCR, 21 were subjected to sequencing confirmation, including 2 HCoV-229E, 9 HCoV-OC43, 9 HCoV-NL63, and 1 HCoV-HKU1. The sequencing reactions were performed by Shanghai Biosune Biotechnology Co., Ltd. (Shanghai, China).

## Figures and Tables

**Table 1:** Primer information for the detection of four HCoVs based on melting curve analysis.

Coronavirus	Primer Set	Primer Name	Sequence (5′-3′)	Amplicon Size (bps)	Theoretical Tm (°C)	Actual Tm (°C)
HCoV-229E	set1	229E-F	TGAAGATGCTTGTACTGTGGCT	513	84.16	83.39
		229E-R	CTGTCATGTTGCTCATGGGG			
	set2	229E-F2	AGATGCTTGTACTGTGGCTTCT	506	84.15	83.48
		229E-R2	CATGTTGCTCATGGGGGAGC			
	set3	229E-F3	TGCTTGTACTGTGGCTTCTTTG	505	84.11	83.52
		229E-R3	GTCATGTTGCTCATGGGGGAG			
HCoV-OC43	set1	OC43-F	ATGTCAATACCCCGGCTGAC	191	84.97	83.96
		OC43-R	GGCTCTACTACGCGATCCTG			
	set2	OC43-F2	CTATCTGGGAACAGGACCGC	122	82.46	83.24
		OC43-R2	TTGGGTCCCGATCGACAATG			
	set3	OC43-F3	ATTGTCGATCGGGACCCAAG	132	82.58	82.32
		OC43-R3	TGTGCGCGAAGTAGATCTGG			
HCoV-NL63	set1	NL63-F	GATAACCAGTCGAAGTCACCTAGTTC	255	81.84	81.10
		NL63-R	ATTAGGAATCAATTCAGCAAGCTGTG			
HCoV-HKU1	set1	HKU1-F	AGGCTCAGGAAGGTCTGCTT	261	81.44	80.00
		HKU1-R	TTAGGAGTTCGCTTTTGGCGA			
	set2	HKU1-F2	AGAGGCAGAAAAACCCAACCTA	374	83.04	83.05
		HKU1-R2	TCCCTTGACGAAACATCGGA			
	set3	HKU1-F3	AGGCAGAAAAACCCAACCTAA	371	83.00	82.75
		HKU1-R3	CCCTTGACGAAACATCGGAG			

HCoVs: The human coronaviruses; Tm: melting temperature. Underlines indicate the primer sets that were selected in the detection group.

## Supplementary Materials

Supplementary materials can be found at www.mdpi.com/1422-0067/17/11/1880/s1.
