# Supplementary material for: A Melting Curve-Based Multiplex RT-qPCR Assay for Simultaneous Detection of Four Human Coronaviruses
Source: Int J Mol Sci. 2016 Nov 23;17(11):1880. doi: 10.3390/ijms17111880 (PMC5133880; doi:10.3390/ijms17111880)
Supplement: Supplementary file 1 [file ijms-17-01880-s001.pdf]

Supplemental Materials: A Melting Curve-Based Multiplex RT-qPCR Assay for Simultaneous Detection of Four Human Coronaviruses

Zhenzhou Wan, Ya'nan Zhang, Zhixiang He, Jia Liu, Ke Lan, Yihong Hu and Chiyu Zhang

Supplemental Results: Optimization of the Primer Concentration

In the conventional PCR, primer concentration was 0.2 μM. When 0.2 μM each set of primers were used to amplify a mixed template containing 30,000 copies of each HCoV RNA, only low or no melting peaks were observed for HCoV-229E, -NL63, and -HKU1, suggesting a competitive amplification of HCoV-OC43 with other three HCoVs. To obtain well performance of the melting curve-based multiplex RT-qPCR, we optimized the primer inputs for HCoV-OC43 and other three HCoVs by orthogonal experiments with primer concentration from 0.2 to 0.6 μM. When GC-HCoV-OC43-F/R was remained at lower concentration of 0.2 μM, the melting peaks of HCoV-229E, -NL63, and -HKU1 were detectable although they were obviously lower than the melting peak of HCoV-OC43 (Figure S2A). In particular, the peaks of HCoV-229E, -NL63, and -HKU1 increased along with the increase of primer concentration. When the concentrations of primers for HCoV-229E, -NL63, and -HKU1 were increased to 0.8 μM, the melting peaks for four HCoVs appeared to have similar peak heights (Figure S2B). Therefore, 0.8, 0.8, 0.8 and 0.2 μM of HCoV-229E, -NL63, -HKU1, and -OC43 specific primers were selected for the assay, respectively.

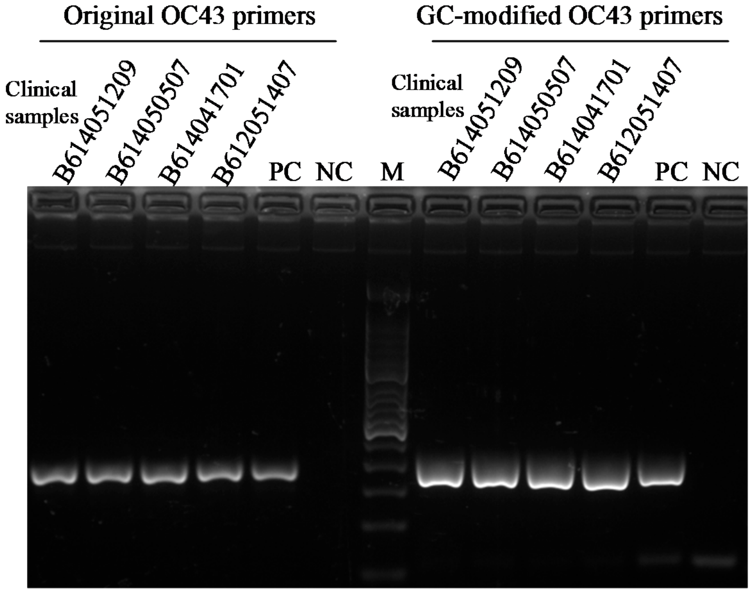

Figure S1. Comparison of the amplification effects between the original OC43 primers and the GC-modified OC43 primers. PC: positive control (OC43 standard strain VR-1558); NC: negative control; M: Marker.

A

Primer concentration (μM for each)	①	②	③	④	⑤	⑥	⑦	⑧	⑨	⑩
229E-F/R, HKU1-F/R, and NL63-F/R	0.6	0.6	0.6	0.6	0.5	0.5	0.5	0.4	0.4	0.3
GC-hCoV-OC43-F/R	0.5	0.4	0.3	0.2	0.4	0.3	0.2	0.3	0.2	0.2

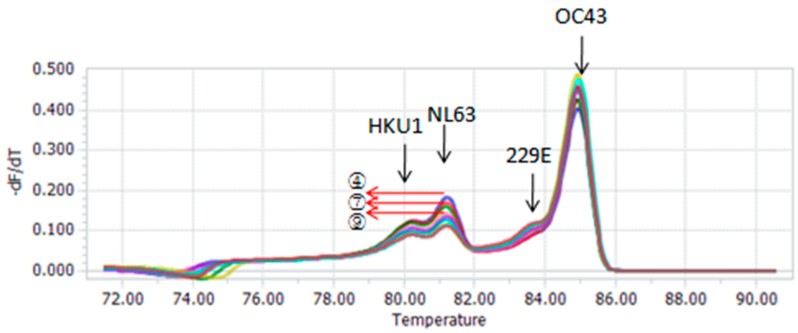

B

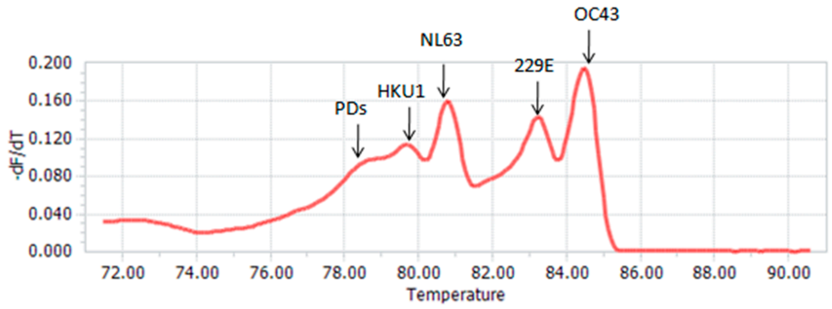

Figure S2. Optimization of the primer concentrations for the multiple RT-qPCR assay. (**A**) Optimization of the multiple RT-qPCR assay using various concentrations of GC-modified HCoV-OC43 and other three HCoVs' primers; (**B**) Melting peaks of four HCoVs using the multiple RT-qPCR assay under optimized primer concentrations. PDs: primer dimers.

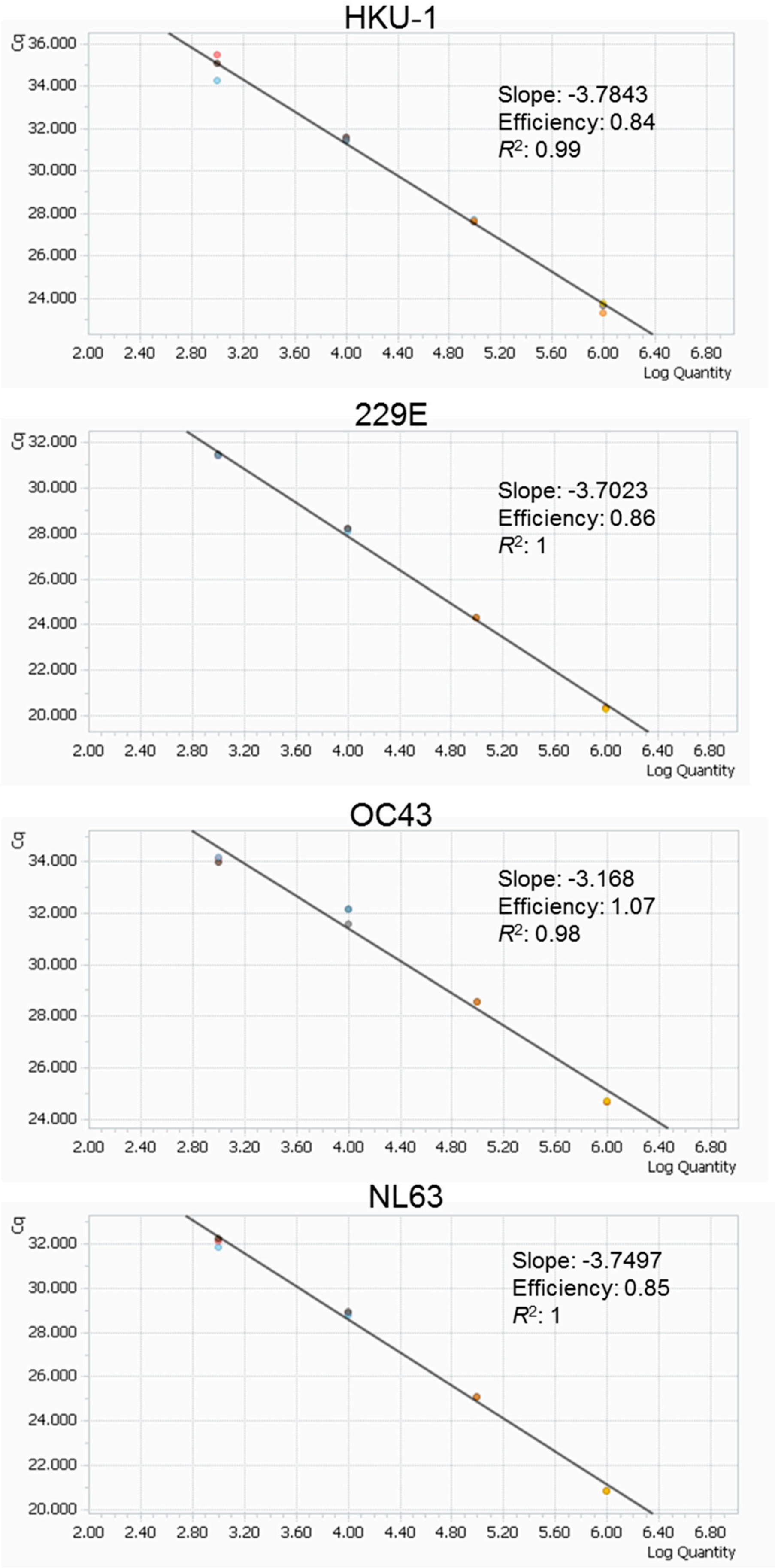

Figure S3. Standard curves of four HCoVs using the multiple RT-qPCR assay.

A (HCoV-NL63)

```
NL63-18   ATCTGAATCC TCCCA--TAT TGTGATTAAA ATCACGAGGA CCAAAGCACT GAATAACATT TTCCTCTCTG GTAGGAACAC GCTTCCAACG AGGTTTCTTC
NL63-19   ........-. -....--... .......... .......... .......... .......... .......... .......... .......... ..........
NL63-20   .......... -....--... .......... .......... .......... .......... .......... .......... .......... ..........
NL63_53   .......... -....--... .......... .......... .......... .......... .......... .......... .......... ..........
NL63-17   TC......T. -...CAT... .......... .......... .......... .......... .......... .......... .......... ..........
NL63-21   .......... -....--... .......... .......... .......... .......... .......... .......... .......... ..........
NL63-22   .......... -....--... .......... .......... .......... .......... .......... .......... .......... ..........
NL63-23   .......... -....--... .......... .......... .......... .......... .......... .......... .......... ..........
NL63-24   .......... -....--... .......... .......... .......... .......... .......... .......... .......... ..........

          AACTGAGAAG GCTTATCAGC CCTAGATTGA GCAAGAGGCT TATTAGGTTT CTTAGGAGTG GAAGTACCAG AAGAACTAGG TGACTTCCAC TGG-TTATC
          .......... .......... ...G.G.... .A........ .G........ .......... .......... .......... .......... ...-.....
          .......... ........C. ...G.G.... .A........ .......... .......... .........T ..C....... .......... ...-.....
          .......... .......... ...G.G.... .A........ .......... .......... .......... .......... .......... ...-.....
          .......... .......... ...G.G.... .A........ .G........ .......... .......... .......... .......... ...-.....
          .......... ........C. ...G.G.... .A........ .......... .......... ........TT ..A....... .......TT. ..TCC...A
          .......... ........C. ...G.G.... .A........ .....C.... .......... .......GTT TTC.....CT ........T. ..T-CCT..
          .......... ........C. ...G.G.... .A........ .......... .......... ........TT .......... .......... ...-.....
          .......... ........C. ...G.G.... .A........ .......... .......... .......GTT TTCC...-CT ........T. A.T-CCT..
```

B (HCoV-OC43)

```
OC43_30   ACGTGCGCGA TGTCAATACC CCGGCTGACA TTGTCGATAG GGACCCAAGT AGCGATGAGG CTATTCCGAC TAGGTTTCCG CCTGGTACGG TACTCCCTCA GGGTTACTAT
OC43_38   ---------. .......... .......... .......... .......... .......... .......... .......... .......... .......... ..........
OC43_49   ---------. .......... .......... ........C. .......... .......... .......... .......... .....C.... .......... ..........
OC43_52   .......... .......... .......... .......... .......... .......... .......... .......... .......... .......... ..........
OC43_55   .......... ....T-.... ....T...-G ...A.A.GC. ....T-GG.. .......... ......G... .......... A...TC.... .......... T.........
OC43_85   .......... .......... .......... ........C. .......... .......... .......... .......... .......... .......... ..........
OC43_86   .......... .......... .......... ........C. .......... .......... .......... .......... .......... .......... ..........
OC43_51   .......... .......... .......... .......... .......... .......... .......... .......... .......... .......... ..........
OC43_54   .......... .......... .......... ........C. .......... .......... .......... .......... .....C.... .......... ..........

          ATTGAAGGCT CAGGAAGGTC TGCTCCTAAT TCCAGATCTA CTTCGCGCAC ATCCAGCAGA GCCTCTAGTG CAGGATCGCG TAGTAGAGCC ACCGCCTGG
          .......... .......... .......... .......... .......... .......... .......... .......... .......... ---------
          .......... .......... .......... .......... .......... .......... .......... .......... .......... ---------
          .......... .......... .......... .......... .......... .......... .......... .......... .......... .........
          G......... .......... .......... .......... .......... ......GTC. .......... .......... ....T..... .........
          .......... .......... .......... .......... .......... .......... .......... .......... .......... .........
          .......... .......... .......... .......... .......... .......... .......... .......... .......... .........
          .......... .......... .......... .......... .......... .......... .......... .......... .......... .........
          .......... .......... .......... .......... .......... .......... .......... .......... .......... .........
```

C (HCoV-229E)

```
229E-5    GTACGTAGAA ACCCATCCTG TGCTATTTTG AGAATTTACG GACCTTCCGA CTCTACTATA AATTATAGTA GTGCTAGGCA CGGCAACTGT CATGTATTCA GGTAGGTTAT
229E-11   .......... .....G.... .......... .......... .......... .......... .......... .....C.... .......... .......... ..........

          GAACCTGAAC ACCTGAAGCC AATCTATGTC CGTCAACGTA AAGCACGCCG CTCAACAAGG TCACAGTAAT GCCTGTTGGA GCTTGTTGAA TGGGTTGATA GTATGTCTGT
          .......... .......... .......... .......... .......... .......... .......... .......... .......... .......... ..........

          CCCAATTACT GT-------G TGATTG--TT GAGCGGCGTG CTTTACGATG -AATGCATAA ATTGTCTTCC AGTGTT-GAA AAGTCTGAAA CTATTTAATA CATGAATCTT
          .....-C..G ..TGTGACA. ......CA.. A.-------- .C.C.G...T CC....CC.. .AA..TCGAG CAC..CG... .......... ......GCA. AG.AC...AC

          CCCCTGAACA AGTGTTGATT CAGTTATAAT GAAGCCAAAT GGTCCAAAGG CCCAATTAAA ATCCCAATTA GCCCAGGTGT CAAAGATTGA CAAAACAAGT ACAATTGGCC
          ..A.AT.... .........A .G.CC....G .....T.... .CAA...... .......... G......... .......... .......... ....G..... ....G.....

          CACGAAGCCC CCCCTACAAG CACCTTCCAA A
          A.A....-.. A.AG...... ..T.C.T... .
```

Figure S4. Alignments of obtained sequences from 9 HCoV-NL63 (**A**); 9 HCoV-OC43 (**B**) and 2HCoV-229E (**C**) positive samples. A dot (.) denotes a base identical to the reference, and a dash (–) denotes a gap.

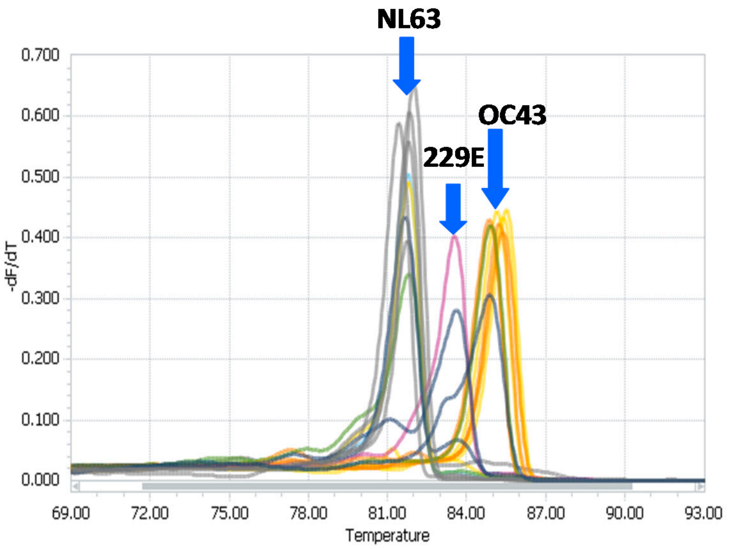

Figure S5. Melting curves of 9 HCoV-OC43, 2 HCoV-229E, and 9 HCoV-NL63 positive samples subjected to sequencing.
